# The Effect of Pulsed Electric Fields (PEF) Combined with Temperature and Natural Preservatives on the Quality and Microbiological Shelf-Life of Cantaloupe Juice

**DOI:** 10.3390/foods10112606

**Published:** 2021-10-28

**Authors:** Li Li, Ruijin Yang, Wei Zhao

**Affiliations:** State Key Laboratory of Food Science and Technology, School of Food Science and Technology, Jiangnan University, Wuxi 214122, China; lili.zz@jiangnan.edu.cn (L.L.); yrj@jiangnan.edu.cn (R.Y.)

**Keywords:** pulsed electric fields, *Saccharomyces cerevisiae*, temperature, natural preservatives, cantaloupe juice, physicochemical characteristics, shelf life

## Abstract

Pulsed electric field (PEF) is an innovative, non-thermal technology for food preservation with many superiorities. However, the sub-lethally injured microorganisms caused by PEF and their recovery provide serious food safety problems. Our study examined the effects of pH, temperature and natural preservatives (tea polyphenols and natamycin) on the recovery of PEF-induced, sub-lethally injured *Saccharomyces cerevisiae* cells, and further explored the bactericidal effects of the combined treatments of PEF with the pivotal factors in cantaloupe juice. We first found that low pH (pH 4.0), low temperature (4 °C), tea polyphenols and natamycin inhibited the recovery of injured *S. cerevisiae* cells. Then, the synergistic effects of PEF, combined with cold-temperature storage (4 °C), a mild treatment temperature (50 and 55 °C), tea polyphenols or natamycin, on the inactivation of *S. cerevisiae* in cantaloupe juice were evaluated. Our results showed that the combination of PEF and heat treatment, tea polyphenols or natamycin enhanced the inactivation of *S. cerevisiae* and reduced the level of sub-lethally injured cells. Moreover, PEF combined with 55 °C heat treatment or tea polyphenols was applied for cantaloupe juice. In the practical application, the two combined PEF methods displayed a comparable inactivation heat pasteurization ability, prolonged the shelf life of juice compared with PEF treatment alone, and better preserved the physicochemical properties and vitamin C levels of cantaloupe juice. These results provide valuable information to inhibit the recovery of PEF-injured microbial cells and shed light on the combination of PEF with other factors to inactivate microorganisms for better food preservation.

## 1. Introduction

There is a growing interest in the applications of non-thermal technologies in food processing and preservation, which display a good capacity for microbial inactivation and can save the natural nutritional and sensory properties of foods [1,2,3]. The pulsed electric field (PEF), as an innovative non-thermal technology, has been investigated in a wide range of fields, including food preservation, undesirable enzyme deactivation and an improvement in juice extraction yield [1,4,5]. It exhibits many advantages for food preservation and quality improvement, especially for the preservation of liquid foods containing heat-sensitive nutrients [4,5]. PEF can inactivate spoilage and pathogenic microorganisms in foods with a low temperature and short processing time, proving to be a better pasteurization technology than traditional thermal pasteurization [1,4].

Sub-lethal injury of foodborne pathogens can be induced by different treatments, e.g., freezing and thawing, ohmic heating, slightly acidic electrolyzed water and PEF [6,7,8,9], and their recovery causes serious food safety concerns. Electroporation of microbial cell membranes has been regarded as a main reason for microbial inactivation by PEF treatment [7]. The impact of PEF on microbial inactivation depends on the process parameters (e.g., electric field intensity, pulse width and treatment time), product parameters (e.g., composition and pH), and microbial characteristics (e.g., type and size) [7,10]. The percentage of cells with reversible or irreversible electroporation (sub-lethal and lethal effects) varied under different conditions, and sub-lethally injured cells can recover under appropriate situations [7,11]. The repair of sub-lethal membrane damage in *Escherichia coli* cells after PEF treatment required energy and lipid synthesis [12]. Moreover, the medium pH and composition played important roles in the recovery of sub-lethally injured *Saccharomyces cerevisiae* cells after PEF treatment [13]. Knowledge of the factors affecting the recovery of sub-lethally injured microorganisms will enable us to find better bactericidal methods. However, our understanding of the factors influencing the recovery of sub-lethally injured microbial cells after PEF treatment is still limited.

Recently, the combinations of different technologies for food preservation have been investigated widely. PEF has been combined with temperature, the addition of antimicrobials, ultrasound and other technologies to promote its microbial inactivation capacity [7,14,15,16,17]. However, the combination of PEF with natural preservatives tea polyphenols or natamycin has not been reported. Our study first used the yeast *S. cerevisiae* as a model organism to examine the impact of pH, temperature and natural preservatives on the recovery of sub-lethally injured cells induced by PEF treatment. Then, the combined treatments of vital factors with PEF were given to the cantaloupe juice, and their bactericidal effects and influences on juice quality were evaluated. The study will provide a great insight into the design of effective microbial inactivation approaches by combining PEF and other food-processing techniques.

## 2. Materials and Methods

### 2.1. Microorganism and Cultivation

The yeast *Saccharomyces cerevisiae* BY4742 (MATalpha, leu2D0, ura3D0, his3D1, lys2D0) was purchased from Open Biosystems (Huntsville, AL, USA). *S. cerevisiae* was first cultured on yeast extract peptone dextrose (YPD) agar slant. Then, one colony was picked and inoculated into 100 mL sterile YPD broth, which was incubated at 30 °C for 14 h with shaking at 200 r/min. The yeast culture was centrifuged at 9000 r/min for 5 min at 4 °C, and the cells were washed twice by the citrate–phosphate buffer. Finally, the cells were resuspended in the citrate–phosphate buffer of pH 7.0 and 4.0, with a final concentration of 10^7^ colony-forming units (CFU)/mL.

### 2.2. Bench Scale PEF Processing System

The PEF equipment used in this study has been described in our previously published work [18]. The PEF treatments were carried out in a bench-scale continuous PEF system (OSU-4A, Ohio State University, Columbus, OH, USA). Six co-field flow tubular chambers with a 2.92 mm electrode gap and a 2.3 mm inner diameter were grouped into three pairs, and each pair was connected with a stainless-steel tubing with a 2.3 mm inner diameter. A model 9310 trigger generator (Quantum Composer, Bozeman, MT, USA) was used to control frequency, pulse duration and delay time between opposite polarities. A cooling coil was connected to each pair of chambers and submerged in a cold-water bath.

### 2.3. PEF Treatment

For the experiment to explore the effects of the electric field intensity and treatment time of PEF on the sublethal injury of *S. cerevisiae*, electric field intensity 15, 20, 25 and 30 kV/cm were applied for 400 μs, and treatment times of 200, 400, 600 and 800 μs were tested with electric field intensity 20 kV/cm. Bipolar square wave pulses (width, 2 μs; pulse repetition rate, 200 Hz) were applied with an energy input ranging from 0.73 to 2.93 kJ/L. For the cell recovery experiments, the cell suspension of *S. cerevisiae* in a citrate–phosphate buffer (10^7^ CFU/mL) was subjected to PEF processing at an electrical field strength of 20 kV/cm for 200 μs, to attain more than 95% sub-lethally injured cells. The pulse repetition rate and pulse width were set at 200 Hz and 2 μs, respectively. During treatment, the water bath temperature was maintained at 5 °C, ensuring that the sample temperature was lower than 20 °C.

### 2.4. Viable Counts

Sub-lethally injured cells are commonly estimated by plating samples to two types of culture medium: a non-selective one (injured cells can repair and recover) and a selective one (injured cells cannot repair and recover). The sub-lethally injured cells can be calculated by the difference in the number of CFU obtained in both mediums. One milliliter of each sample was serially diluted in sterile normal saline. Then, 1 mL of the appropriate dilution was plated on 15 mL YPD (non-selective medium), and 1 mL was plated on the YPD medium with 7% sodium chloride (YPD-SC, selective medium). According to our previous experiment, 7% sodium chloride can inhibit the recovery and growth of injured cells, while the untreated cells were able to grow. Plates with YPD medium were incubated for 48 h at 30 °C and those with YPD-SC medium were incubated for 96 h at the same temperature. It has been shown that the longer incubation time did not affect viable counts). The level of inactivation was expressed as log_10_ (N_0_/N) or log_10_ (N/N_0_) (survival fraction). N_0_ refers to the bacterial counts before the PEF treatment, whereas N refers to the bacterial counts after the PEF treatment. The recovery of injured cells was defined as an increase in bacterial counts on the selective medium, while no increase was observed on the non-selective medium.

### 2.5. Recovery of the Sublethally Injured Cells under Different Conditions

The PEF-treated *S. cerevisiae* suspensions were incubated at 30 °C for 4 h, at pH 4.0 and 7.0, without or with the addition of natural preservatives, to evaluate the effect of pH and natural preservatives on the recovery of injured cells. To examine the impact of temperature on the recovery of injured cells, the cell suspensions in a citrate–phosphate buffer were also incubated at 4 °C for 4 h. The natural preservatives used were tea polyphenols (400 mg/kg) and natamycin (300 mg/kg) (Yuanye Bio-Technology, Shanghai, China). Both products meet the microbiological requirements. Tea polyphenols were first dissolved in sterile deionized water with a high concentration (100 g/L), and then the solution was sterilized by filtration with the 0.22-μm filter. The sterile tea polyphenols solution was used for future treatments (the addition of tea polyphenols), while natamycin, as an effective antibacterial agent, was directly used. Their concentrations were determined based on the National Food Safety Standard: Standards for Uses of Food Additives (GB2760-2011) and the content of tea polyphenols in tea drinks that were available on the market. Samples were taken at preset intervals (timepoints: 0, 10 min, 30 min, 1 h, 2 h and 4 h) to measure viable counts, which were evaluated by plating samples on YPD and YPD-SC, as described above.

### 2.6. The Inactivation of S. cerevisiae in Cantaloupe Juice

Cantaloupes were purchased from a local fruit market (Wuxi, China). Cantaloupes were peeled and sliced, then juiced by a juice extractor (YZ-V15, Joyoung Company Limited, Jinan, China). Then, the extracted juice was filtered by vacuum suction filtration. The electrical conductivity of the cantaloupe juice was adjusted to 2000 μS/cm by adding sterile deionized water. The *S. cerevisiae* cells were suspended in the cantaloupe juice with a final concentration of 10^7^ CFU/mL. The cantaloupe juice with *S. cerevisiae* was treated by PEF at 20 kV/cm for 200 μs. After PEF treatment, the cantaloupe juice was stored at 4 °C for 7 days, or incubated in a water bath at 50 °C/55 °C for 5 min and subsequently stored at 4 °C for 7 days, to examine the synergistic effect of PEF with temperature on *S. cerevisiae* inactivation. For the mild heat treatment, the PEF-treated solutions passed through a coiled tube with a 2.3 mm inner diameter, which was submerged in two water baths (the first is for pre-heating and the second is for incubation). To explore the synergistic effect of PEF and natural preservatives, the sterile tea polyphenols solution or natamycin was added to cantaloupe juice before or after PEF treatment. The final concentration is 400 mg/kg for tea polyphenols and 300 mg/kg for natamycin. Before storage, the untreated and treated cantaloupe juices were aseptically packaged into 200 mL sterile polypropylene bottles (without headspace) in a laminar airflow cabinet.

### 2.7. The Analysis of Physicochemical Characteristics of Cantaloupe Juice

The fresh cantaloupe juice was treated with different bactericidal methods, including 90 °C for 3 min, PEF 30 kV/cm for 400 μs, 400 mg/kg tea polyphenols addition and PEF 30 kV/cm for 400 μs, PEF 20 kV/cm for 400 μs and then 55 °C for 5 min. The electrical conductivity, pH, total soluble solids, color changes and vitamin C in untreated and treated juices were determined. The electrical conductivity, pH and total soluble solid amount of the juice were measured by a conductivity meter (Dd-11c, Shanghai Precision Scientific Instrument Co., LTD, Shanghai, China), a pH meter (Mettler Toledo, Shanghai, China) and a digital refractometer (Atago, Tokyo, Japan), respectively. Color analysis was conducted on a calibrated Hunter Lab colorimeter (Hunter Lab, Reston, VA, USA). Color measurements (L*, a* and b* values) were determined. The color difference was calculated as ΔE = [(L* − L_0_*)^2^ + (a* − a_0_*)^2^ + (b* − b_0_*)^2^]^1/2^, where L_0_*, a_0_* and b_0_* were the values of the untreated sample. The amount of vitamin C (ascorbic acid) in the cantaloupe juice was determined by the reduction in the blue dye 2,6-dichloroindophenol [19].

### 2.8. Microbial Analysis

The total aerobic bacteria in the cantaloupe juice were determined by the plate count agar (incubated at 36 °C for 2 days). The yeast and mold counts were determined by the potato dextrose agar (incubated at 28 °C for 5 days). Results were expressed as log_10_ CFU/mL.

### 2.9. Statistical Analysis

Each experiment was carried out in triplicate. Data in each figure are presented as mean ± standard deviation. One-way analysis of variance (ANOVA) and Tukey’s multiple comparison test were conducted with SAS software (Version 9.2, Cary, NC, USA). The significance level was 5% in all analyses.

## 3. Results and Discussion

### 3.1. Effects of Electric Field Intensity and Treatment Time of PEF on the Sublethal Injury of S. cerevisiae

**Figure 1:**
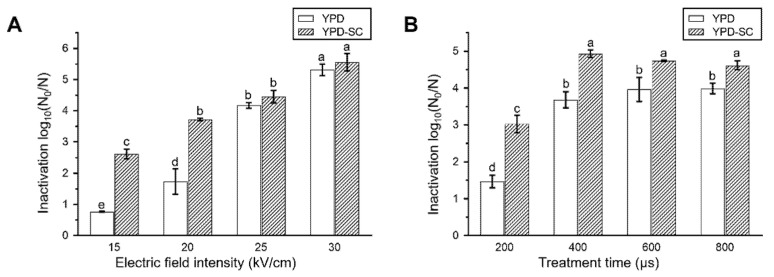
Effects of different electric field intensity (**A**) and treatment time (**B**) of PEF on inactivation and sublethal injury of *S. cerevisiae*. YPD, non-selective medium; YPD-SC, selective medium. Data are expressed as mean ± standard deviation. Significant differences are indicated by different letters (*p* < 0.05).

We first showed that different amounts of sub-lethally injured cells were generated after exposure to diverse electric field intensity (15, 20, 25 and 30 kV/cm) and treatment time (200, 400, 600 and 800 μs) of PEF. As shown in Figure 1, more sub-lethally injured cells were produced when the electric field intensity was set as 15 and 20 kV/cm with the same treatment time (400 μs) (Figure 1A). The most sub-lethally injured cells were produced when the treatment time was 200 μs with 20 kV/cm intensity (Figure 1B). With the increase in electric field intensity or treatment time, the number of sub-lethally injured cells decreased, and the inactivation effect became stronger. Thus, 20 kV/cm and 200 μs of PEF treatment were chosen for the following experiments to generate a large amount of sub-lethally injured cells and study their recovery characteristics under different conditions.

### 3.2. The Effect of pH and Temperature on the Recovery of PEF-Injured S. cerevisiae

**Figure 2:**
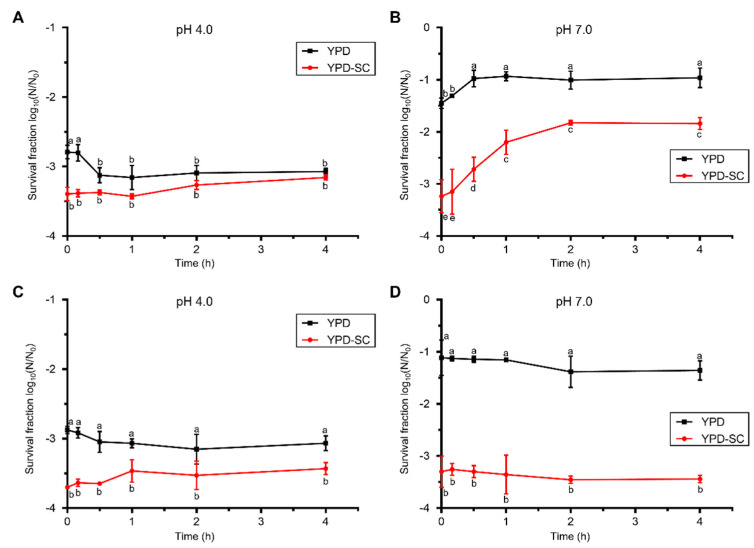
The effect of pH and temperature on the recovery of PEF-injured *S. cerevisiae*. Survival fraction of *S. cerevisiae* after the PEF treatment (20 kV/cm for 200 μs) in citrate-phosphate buffer at pH 4.0 (**A**,**C**) and pH 7.0 (**B**,**D**), and in the subsequent recovery process incubated at 30 °C (**A**,**B**) or 4 °C (**C**,**D**) for 4 h. YPD, non-selective medium; YPD-SC, selective medium. Data are expressed as mean ± standard deviation. Significant differences are indicated by different letters (*p* < 0.05).

*S. cerevisiae* cells were treated by PEF at 20 kV/cm for 200 μs in a citrate–phosphate buffer with pH 4.0 and 7.0, which generated a high number of sub-lethally injured cells. To examine the effect of pH on the recovery of injured *S. cerevisiae* cells after the PEF treatment, the PEF-treated cells were incubated in citrate–phosphate buffer with different pHs for 4 h. As shown in Figure 2, viable counts were reduced by about 2.8 log_10_ and sub-lethally injured cells were generated by less than 1.0 log_10_ after PEF treatment at pH 4.0 (Figure 2A). The inactivation of *S. cerevisiae* was 1.4 log_10_ and the occurrence of sub-lethally injured *S. cerevisiae* was 1.8 log_10_ at pH 7.0 with the same PEF treatment (Figure 2B). More *S. cerevisiae* cells were killed at pH 4.0 and a larger proportion of sub-lethally injured cells was produced at pH 7.0. The results indicate that *S. cerevisiae* cells are sensitive to a low pH, and the PEF resistance of the tested strain depends on the pH of the treatment medium. However, the amounts of citric acid and phosphate in the two kinds of medium were different. The distinct inactivation effects may be due to the different amounts of phosphate and citric acid.

The survival fraction of PEF-treated *S. cerevisiae* cells counted by non-selective mediums decreased after 30 min incubation in the citrate–phosphate buffer with pH 4.0, and there was nearly no change based on selective mediums (Figure 2A,C), suggesting the inactivation of the sub-lethally injured cells. A slight recovery in pH 4.0 repair medium was observed from 1 h to the end of the recovery (Figure 2A). For pH 7.0 repair medium, injured cells recovered rapidly in the first two hours and about half of them were repaired (Figure 2B). Additionally, cell regeneration occurred at the pH 7.0 repair medium. Our results are consistent with previous findings that PEF induced sub-lethally injured cells were unable to recover and died in the citrate–phosphate buffer at pH 4.0 [20], and sub-lethally injured yeast cells or *E. coli* cells are sensitive to acids [11,21]. Hence, PEF treatment for foods with a low pH will achieve a prominent pasteurization effect.

The impact of low temperature (4 °C) on the recovery of PEF-injured *S. cerevisiae* was investigated. The nearly horizontal trendlines in Figure 2C,D showed that injured cells incubated at 4 °C after PEF treatment were hardly repaired at both pH values. In contrast, injured cells recovered when they were incubated at 30 °C (Figure 2A,B). Our findings suggest that storage temperature following PEF treatment is a significant factor influencing the repair process of PEF-induced sub-lethally injured cells. The low storage temperature (4 °C) inhibited the recovery of sub-lethally injured microorganisms in PEF-treated green tea infusions and 7-day cold storage after PEF treatment could effectively extend the shelf life of PEF-treated green tea infusions [22]. Garcia et al. [23] demonstrated that PEF-injured *E. coli* O157:H7 cells were sensitive to low temperature. The combination of PEF treatment (25 kV/cm for 400 μs) with 4 °C storage for 48 h inactivated 5 log_10_ *E. coli* in apple juice (pH 3.8). The PEF treatment causes electroporation on microbial cell membranes, and membrane-associated metabolic processes may become slow when the temperature is low, which may obstruct the repair of injured cells.

### 3.3. The Effect of Natural Preservatives on the Recovery of PEF-Injured S. cerevisiae

**Figure 3:**
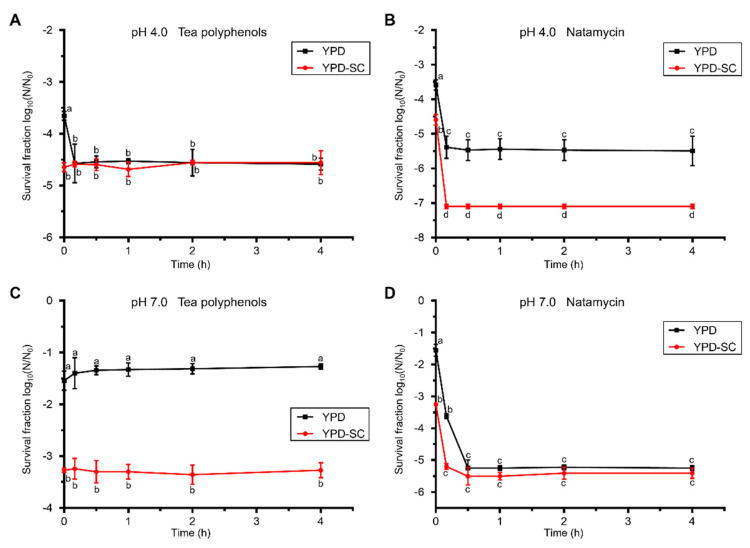
The effect of natural preservatives on the recovery of PEF-injured *S. cerevisiae*. Survival fraction of *S. cerevisiae* after the PEF treatment (20 kV/cm for 200 μs) in citrate–phosphate buffer with pH 4.0 (**A**,**B**) and pH 7.0 (**C**,**D**), and in the subsequent recovery process incubated at 30 °C for 4 h in mediums with tea polyphenols (**A**,**C**) and natamycin (**B**,**D**). YPD, non-selective medium; YPD-SC, selective medium. Data are expressed as mean ± standard deviation. Significant differences are indicated by different letters (*p* < 0.05).

The influence of two natural preservatives on the recovery of PEF-injured *S. cerevisiae* was evaluated by incubating PEF-treated (20 kV/cm for 200 μs) cells in a citrate–phosphate buffer with the addition of tea polyphenols or natamycin at 30 °C for 4 h. The survival fraction of *S. cerevisiae* cells, evaluated based on a selective medium, did not increase in any recovery conditions (Figure 3), suggesting that injured *S. cerevisiae* cells in mediums with natural preservatives were unable to recover.

For the pH 4.0 repair mediums, the survival fraction calculated based on the non-selective medium decreased significantly in the first 10-min incubation with tea polyphenols. In comparison, the survival fraction calculated based on the selective medium kept constant and remained the same as that based on the non-selective medium after 10 min of recovery (Figure 3A). The result suggests that sub-lethally injured cells are sensitive to tea polyphenols and were killed by them, and tea polyphenols had no effect on intact cells. Natamycin showed a better antimicrobial effect than tea polyphenols. The viable counts evaluated by both the non-selective and selective mediums were reduced by more than 5 log_10_ after 10-min incubation with natamycin (Figure 3B).

The survival fraction of *S. cerevisiae* recovered in the citrate–phosphate buffer of pH 7.0 with tea polyphenols remained constant for both non-selective and selective mediums (Figure 3C). This demonstrated that tea polyphenols could inhibit the recovery of sub-lethally injured cells at pH 7.0, but could not further inactivate them and the intact cells. For injured cell recovery in a repair medium at pH 7.0 with natamycin (Figure 3D), both intact and sub-lethally injured cells were killed by natamycin with more than 5 log_10_ reductions after 30 min incubation.

Tea polyphenols are beneficial to human health and used as a natural food additive, because of their antioxidative and antimicrobial actions. Natamycin is another natural bio-preservative used in the food industry of cheese, meat products and beverages. The antimicrobial activities of tea polyphenols and natamycin have been investigated, and both can extend the food’s shelf life [24,25]. Our results showed that tea polyphenols displayed distinct antimicrobial effects on injured *S. cerevisiae* cells at different pH values. Tea polyphenols inhibited injured cells from repairing at pH 7.0, and killed injured cells at pH 4.0. The membrane damage of sub-lethally injured cells after PEF treatment may assist tea polyphenols into cells, which better inactivates cells. Additionally, natamycin had the best antimicrobial effect on *S. cerevisiae* cells in our study, with five log_10_ reductions in viable counts. Previous studies suggest that natamycin may react with sterols on the cell membrane, and then cause cell structure changes and cell permeabilization [25]. The combination of PEF with tea polyphenols or natamycin may provide a synergistic antimicrobial effect and better capacity to inhibit and inactivate microorganisms.

### 3.4. Inactivation of S. cerevisiae in Cantaloupe Juice by Combining PEF with Temperature or Natural Preservatives

**Figure 4:**
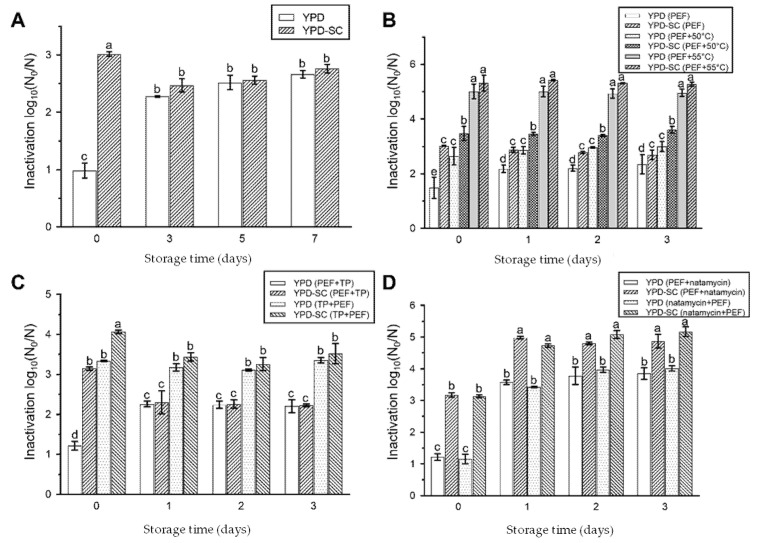
Inactivation of *S. cerevisiae* in cantaloupe juice by combining PEF with temperature or natural preservatives. The synergistic effects of PEF combined with low temperature (4 °C) (**A**), mild temperature (50 and 55 °C) (**B**), tea polyphenols (**C**) and natamycin (**D**) on the inactivation of *S. cerevisiae* cells in cantaloupe juice. Data are expressed as mean ± standard deviation. Significant differences are indicated by different letters (*p* < 0.05).

The above findings showed that temperature and natural preservatives were essential factors to inhibit the recovery of sub-lethally injured *S. cerevisiae* cells. Then, we further examined the synergistic effect of PEF with temperature or natural preservatives on the inactivation of *S. cerevisiae* in cantaloupe juice. A low storage temperature (4 °C), mild treatment temperature (50 and 55 °C), tea polyphenols and natamycin were used in our study. The cantaloupe juice with *S. cerevisiae* was treated by PEF at 20 kV/cm for 200 μs, and then stored at 4 °C for 7 days. As seen in Figure 4A, 1.9 log_10_ sub-lethally injured cells were produced after PEF treatment. After 3 days of cold storage, the inactivation of *S. cerevisiae* cells in a non-selective medium increased from 0.9 log_10_ to 2.3 log_10_, indicating that 1.4 log_10_ sub-lethally injured cells died in cold storage. While the inactivation of *S. cerevisiae* cells in a selective medium decreased from 3.0 log_10_ to 2.5 log_10_, suggesting that 0.5 log_10_ sub-lethally injured cells were repaired in cold storage. The viable counts on both mediums changed slightly for further cold storage and the sub-lethally injured cells tended to disappear. The results demonstrated that low storage temperature is an effective method to inactivate sub-lethally injured microorganisms.

After PEF treatment at 20 kV/cm for 200 μs, the cantaloupe juice with *S. cerevisiae* was incubated in a water bath at 50 °C or 55 °C for 5 min, then stored at 4 °C. The PEF treatment, combined with 50 °C or 55 °C, enhanced the inactivation of *S. cerevisiae*, especially for treatment combined with 55 °C, which had an over 5.0 log_10_ inactivation (Figure 4B). The sub-lethally injured cells were decreased from 1.5 log_10_ (only PEF treatment) to 0.8 log_10_ (PEF treatment combined with 50 °C) and 0.3 log_10_ (PEF treatment combined with 55 °C). The number of live and sub-lethally injured cells in cantaloupe juice after the combined treatments did not change significantly in the cold storage. Our results showed that the combination of PEF and heat treatment could not only improve the *S. cerevisiae* inactivation, but also further reduced the number of sub-lethally injured cells. Sub-lethally injured cells may be killed by mild heat treatment, because of their damaged cell membrane.

The effects of PEF treatment combined with tea polyphenols or natamycin, and the treatment sequence, on the inactivation of *S. cerevisiae,* were evaluated. The synergistic sequence of PEF and tea polyphenols had a significant impact on the inactivated and sublethally injured *S. cerevisiae* cells (Figure 4C). When PEF treatment was performed first, 1.2 log_10_ cells were inactivated and 1.9 log_10_ cells were sub-lethally injured. When PEF treatment was performed after the addition of tea polyphenols, 3.4 log_10_ cells were inactivated and only 0.7 log_10_ cells were injured. The addition of tea polyphenols before PEF treatment could promote the inactivation effect. In the cold storage, the two combined treatments displayed similar changing trends. That is, the number of sub-lethally injured cells decreased, due to their death or recovery. The synergistic sequence of PEF and natamycin had no significant effect on the inactivated and sub-lethally injured *S. cerevisiae* cells (Figure 4D), different from that of tea polyphenols. The difference between tea polyphenols and natamycin treatments may be related to their distinct antibacterial mechanisms. Tea polyphenols may inactivate bacteria by damaging cell membranes and causing metabolic disorders by targeting microbial enzymes [26]. The membrane electroporation caused by PEF treatment might better facilitate the entrance of tea polyphenols into the cell to conduct their function. However, the effect of natamycin on bacteria inactivation may be due to its reaction with sterols on the cell membrane, leading to changes in cell membrane structure and permeability [25].

### 3.5. The Effect of PEF-Combined Treatments on the Quality and Microbiological Shelf-Life of Cantaloupe Juice

PEF, in combination with temperature or natural preservatives, showed an enhanced capacity to inactive *S. cerevisiae* and inhibit the repair of the injured cells in cantaloupe juice. Thus, the effects of these methods on the quality and microbial indexes of cantaloupe juice were further evaluated for their practical application. Four bactericidal methods were investigated and compared in our study. We chose these four methods because they showed similar bactericidal effects. As shown in Table 1, no treatments affected the pH of cantaloupe juice. The electrical conductivity and total soluble solids in cantaloupe juice treated by heat pasteurization (90 °C for 3 min) significantly increased compared with the untreated cantaloupe juice. However, PEF-related treatments did not affect these parameters. The color of cantaloupe juice significantly changed (ΔE > 2, *p* < 0.05) with the treatment of heat pasteurization or PEF combined with tea polyphenols. The color difference (ΔE) was the highest in cantaloupe juice treated with PEF combined with tea polyphenols, which may be due to the color of tea polyphenols. All treatments caused the loss of vitamin C, but only heat pasteurization caused the significant decrease in vitamin C in cantaloupe juice (Table 1). The treatment of PEF combined with tea polyphenols saved the most vitamin C. Our results showed that PEF and PEF-combined treatments could better preserve the physicochemical characteristics and nutritional components of cantaloupe juice. Many studies have demonstrated the advantages of PEF pasteurization in the preservation of product physicochemical properties [27,28,29,30]. For example, PEF processing cannot cause significant reductions in total phenolic, anthocyanin, and antioxidant compounds in pomegranate juice compared with untreated juice; the total phenolics and total carotenoids of grapefruit juice have even been shown to increase after PEF treatment [28]. Compared with the thermal pasteurization, PEF processing saved more flavonoids, phenolic acids and volatile compounds of orange and apple juices; the organoleptic characteristics (e.g., color, taste, flavor and overall acceptance) were better preserved in the PEF-treated juices [28]. The effect of PEF treatment and its combination with tea polyphenols on more nutritional components and organoleptic characteristics of cantaloupe juice should be further studied.

**Figure 5:**
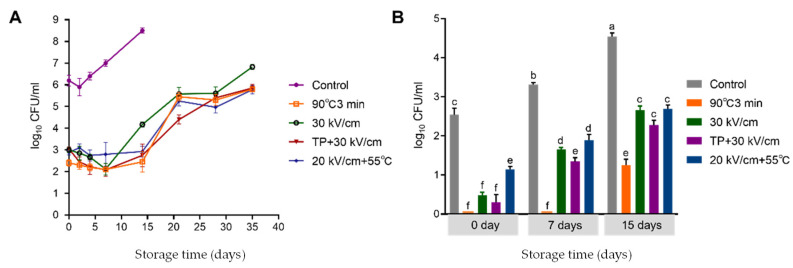
Microbial changes in PEF-treated cantaloupe juice stored at 4 °C. (**A**) Changes of total aerobic bacteria in PEF-treated cantaloupe juice stored at 4 °C. (**B**) Changes in yeast and mold in PEF-treated cantaloupe juice stored at 4 °C. Data are expressed as mean ± standard deviation. Significant differences are indicated by different letters (*p* < 0.05). Control, untreated cantaloupe juice; 90 °C, treated at 90 °C for 3 min; 30 kV/cm, treated at 30 kV/cm for 400 μs; TP + 30 kV/cm, treated with 400 mg/kg tea polyphenols and then treated at 30 kV/cm for 400 μs; 20 kV/cm + 55 °C, treated at 20 kV/cm for 400 μs and then treated at 55 °C for 5 min.

The changes in total aerobic bacteria, yeast and mold in cantaloupe juice were investigated after PEF treatments and during storage. The initial total number of aerobic bacteria in untreated cantaloupe juice was more than 6.0 log_10_, which decreased to about 3.0 log_10_ after inactivation by different methods (Figure 5A). Total aerobic bacteria in samples treated with PEF was kept at a low level during the first seven days’ storage, which increased rapidly after 7 days. However, the total aerobic bacteria in samples treated by the other methods were kept at low levels within 15 days of cold storage, making the shelf life of cantaloupe juice treated with these methods last more than 15 days (total bacteria <10^5^ CFU/mL, Figure 5A). Additionally, all methods reduced the number of yeast and mold to low levels (<1.2 log_10_) after treatments, and the number of yeast and mold in these treated samples after 15 days’ cold storage was similar to those detected in the untreated fresh cantaloupe juice (Figure 5B).

Our results revealed that the four methods applied in our study displayed good microorganism inactivation effects, and the combination of PEF with tea polyphenols or mild heat performed better than PEF treatment alone. Studies have demonstrated the microbial inactivation effect of PEF treatment for different fruit juices, inactivating the spoilage and pathogenic microorganisms, vegetative bacteria, yeast and mold [5,27]. PEF has also been combined with other techniques, including temperature, the addition of antimicrobials (e.g., nisin and sorbic acid), ultrasound, high pressure and manothermosonication, to enhance its microbial inactivation effects [7,14,15,16,17]. For example, the combination of PEF and ultrasound could preserve bioactive compounds in grapefruit juice and extend its shelf life [17]. However, no studies have examined the bactericidal effect of PEF treatment in combination with tea polyphenols. Tea polyphenols, as natural preservatives, have been shown to have antioxidant, anti-inflammatory and anti-cancer effects, and are beneficial for human health [31,32,33]. The combination of PEF with tea polyphenols showed an excellent bactericidal effect and preserved the quality of cantaloupe juice. The application of PEF combined with tea polyphenols for food preservation could be considered in food processing.

## 4. Conclusions

The sublethal injury of cells caused by PEF treatment is reversible and can be repaired under appropriate conditions. We found that a low pH, low temperature and natural preservatives (tea polyphenols and natamycin) could hinder the recovery of sub-lethally injured *S. cerevisiae* cells. PEF combined with mild heat treatment, tea polyphenols or natamycin boosted the inactivation of *S. cerevisiae* and reduced the number of sub-lethally injured cells in cantaloupe juice. Additionally, in practical applications the combination of PEF with 55 °C heat treatment or tea polyphenols had similar effects on microbial inactivation to thermal pasteurization and extended the shelf life of cantaloupe juice compared with the PEF treatment alone. Moreover, the two PEF-combined methods better preserved the quality of cantaloupe juice. The factors which inhibit the repair process of sub-lethally injured microorganisms after PEF treatment should be considered in food processing. The combination of these factors and PEF provides promising methods for food pasteurization, prolonging the shelf life of food and retaining food’s physicochemical characteristics. Further studies should be conducted to examine the mechanism underlying the synergistic effect of PEF with other factors for microbial inactivation.

## Figures and Tables

**Table 1:** Physicochemical changes of cantaloupe juice after PEF treatment.

Treatments	Electrical Conductivity (μS/cm)	pH	Total Soluble Solids (%)	ΔE	Vitamin C (mg/100 mL)
Control	1821 ± 3.77 ^b^	6.44 ± 0.01 ^a^	2.51 ± 0.01 ^b^	——	1.149 ± 0.02 ^a^
90 °C	1843 ± 4.24 ^a^	6.45 ± 0.00 ^a^	2.85 ± 0.03 ^a^	3.08 ± 0.04 ^b^	0.748 ± 0.01 ^b^
30 kV/cm	1818 ± 5.66 ^b^	6.44 ± 0.02 ^a^	2.50 ± 0.04 ^b^	1.58 ± 0.12 ^d^	0.924 ± 0.01 ^a^
TP + 30 kV/cm	1812 ± 1.41 ^b^	6.45 ± 0.00 ^a^	2.50 ± 0.01 ^b^	5.86 ± 0.00 ^a^	1.013 ± 0.04 ^a^
20 kV/cm + 55 °C	1823 ± 1.41 ^b^	6.45 ± 0.01 ^a^	2.50 ± 0.02 ^b^	1.81 ± 0.07 ^c^	0.997 ± 0.02 ^a^

Data are expressed as mean ± standard deviation. Significant differences within a column are indicated by different letters (*p* < 0.05). Control, untreated cantaloupe juice; 90 °C, treated at 90 °C for 3 min; 30 kV/cm, treated at 30 kV/cm for 400 μs; TP + 30 kV/cm, treated with 400 mg/kg tea polyphenols and then treated at 30 kV/cm for 400 μs; 20 kV/cm + 55 °C, treated at 20 kV/cm for 400 μs and then treated at 55 °C for 5 min.

## Data Availability

The datasets generated for this study are available on request to the corresponding author.
